# Single-port-plus-one robot-assisted laparoscopic Lich–Gregoir direct nipple ureteral extravesical reimplantation in pediatric primary obstructive megaureter, comparing to laparoscopic cohen

**DOI:** 10.1186/s40001-024-01862-z

**Published:** 2024-05-08

**Authors:** Shan Lin, Huihuang Xu, Yufeng He, Xinru Xu, Guangxu You, Jianglong Chen, Di Xu

**Affiliations:** 1 Shengli Clinical Medical College of Fujian Medical University, 1 North Xuefu Road, Fuzhou, 350122 Fujian China; 2 Department of Pediatric Surgery, Fujian Provincial Hospital, Fuzhou, China 134 Dongjie Road ,Gulou District, 350001

**Keywords:** Single-port-plus-one robot-assisted laparoscopic, Primary obstructive megaureter, Lich–Gregoir direct nipple, Cohen

## Abstract

**Purpose:**

To compare the effects of a single-port-plus-one robotic laparoscopic-modified Lich–Gregoir direct nipple approach and traditional laparoscopic Cohen in treating pediatric primary obstructive megaureter.

**Materials and methods:**

The clinical data of 24 children with primary obstructive megaureter from January 2021 to November 2021 were analyzed retrospectively. Among them, 12 children (8 boys and 4 girls, the average age were 17.17 ± 6.31 months) treated with the laparoscopic Cohen method were defined as group C. The remaining 12 children (7 boys and 5 girls, the average age was 17.33 ± 6.99 months) underwent single-port-plus-one robotic laparoscopic-modified Lich–Gregoir direct nipple ureteral extravesical reimplantation were defined as group L. The parameters of pre-operation, intraoperative and postoperative were compared.

**Results:**

There were no differences in the patient characteristics and average follow-up time between the two groups (*P* > 0.05).The obstruction resolution rate was 100% in both groups. The total operation time in group L is slightly longer than that in group C(*P* < 0.001),but the intraperitoneal operation time of the two groups was comparable(*P* > 0.05). The postoperative parameters included blood loss, gross haematuria time, indwelling catheterization time and hospitalization time in group L is shorter than group C(*P* < 0.05). One year post-operation, decreasing in ureteral diameter and APRPD, and increasing in DRF were remarkably observed in both two groups(*P* < 0.05). Ureteral diameter, APRPD, and DRF were not significantly different both in pre-operation and post-operation between Group L and Group C(*P* > 0.05).

**Conclusion:**

Single-port-plus-one robot-assisted laparoscopic-modified Lich–Gregoir direct nipple approach and traditional laparoscopic Cohen are both dependable techniques for ureteral reimplantation in the treatment of pediatric primary obstructive megaureter. Since Lich–Gregoir can preserve the physiological direction of the ureter and direct nipple reimplantation enhances the effect of anti-refluxing, this technique is favorable for being promoted and applied in robot surgery.

## Introduction

Congenital megaureter accounts for 10% of urinary tract diseases in children, and the overall incidence is about 1:1500–1:2000. Antenatal ultrasound diagnosis has important clinical value in the early diagnosis of megaureter. In fact, the diameter of the pelvic ureter ≥ 7mm at 30 weeks of pregnancy is an abnormal manifestation [1]. In 1976, the American Pediatric Association further divided the causes of megaureter disease onto: obstruction type, reflux type, non-obstructive non-reflux type, obstruction, and reflux type [2].The most important therapeutic goal for obstructive megaureter is to remove urinary tract obstruction and protect renal function. Open reimplantation with ureteral tailoring is the standard procedure for the treatment of primary obstructive megaureter. The pneumovesical laparoscopic Cohen approach and extravesical laparoscopic Lich–Gregoir approach are the most frequently used minimally invasive methods. Laparoscopic Lich–Gregoir approach with obvious surgical efficacy and better postoperative recovery was applied in treatment of VUR for decades [3]. The modified technique was also used in treatment of POM. With higher definition, fibrillated-free wrist, robot surgery is suitable for pediatric surgery, especially in pelvic narrow cavity, and is a new trend of MIS [4]. However, there were fewer reports described robot-assisted laparoscopic Lich–Gregoir surgery in POM [5].

In view of this, we introduced the single-port-plus-one robot-assisted laparoscopic-modified Lich–Gregoir direct nipple approach to treat POM, and compared its efficacy and safety with traditional laparoscopic Cohen approach.

## Materials and methods

### General information

The clinical data of 24 children with primary obstructive megaureter admitted to Fujian Provincial Hospital from January 2021 to November 2021 were analyzed retrospectively. Among them, twelve children treated with the laparoscopic Cohen method from January 2021 to June 2021 were defined as group C. The remaining twelve children who underwent single-port-plus-robot-assisted laparoscopic-modified Lich–Gregoir direct nipple approach from June 2021 to November 2021 were defined as group L. There were eight boys and four girls in group C and the average age was 17.17 ± 6.31 months. There were seven boys and five girls in group L and the average age was 17.33 ± 6.99 months. In group L, there were five right-side obstructions and seven left-side obstructions and the types of POM were three obstructions with refluxing and nine obstruction without refluxing. The UTDS grades were seven UTD P2 and five UTD P3. In group C, there were six right-side obstructions and six left side obstructions and the types of POM were two obstruction with refluxing and ten obstruction without refluxing. The UTDS grades were eight UTD P2 and four UTD P3. In group L, prenatal diagnosis was carried out in 12 cases, 2 patients had abdominal pain and UTIs were presented in 3 patients. In group C, there were 9patientsprenatal diagnosed and 1patientsabdominal pain and 2 patients had UTIs. Basic patients’ characteristic in two groups had no statistical significance (Table 1, P > 0.05).

**Table 1:** Basic Patient Information in two group

	Group L	Group C	χ2 (*t*)	P
N (number of patients)	12	12		
Sex				
Male	7	8	0.178	0.673
Female	5	4		
Side of surgery				
Left	7	6	0.186	0.682
Right	5	6		
Average age (months)	17.33 ± 6.99	17.17 ± 6.31	0.61	0.952
The type of POM^a^				
Obstruction with refluxing	3	2	0.253	0.615
Obstruction without refluxing	9	10		
UTD grade^b^				
UTD P2	7	8	0.178	0.673
UTD P3	5	4		

*SD* standard deviation, *POM* primary obstructive megaureter

^a^According to the system of American Pediatric Association for classifying the megaureter

^b^According to urinary tract dilation (UTD) risk stratification

Preoperatively, all patients underwent magnetic resonance urography (MRU) and urological ultrasound to discovery POM, diuretic renogram to evaluate urine drainage curve, renal static imaging (99 m Tc-DMSA) to assess DRF and voiding cystourethrography (VCUG) to find VUR.

All robot-assisted or laparoscopic surgery were performed by the same surgeon and surgical team. There were five cases underwent ureteral tapering repair in group L and four cases in group C, which all were performed in accordance with Hendren technique. Informed consent was obtained from the parents of the child and the study was ethically reviewed by our institution (ethics approval number: K2020-12–033).

### Inclusion and exclusion criteria

Inclusion criteria: POM patients with symptoms such as febrile UTIs or pain, and in the asymptomatic patient, a DRF below 40% associated with massive or progressive hydronephrosis, or a drop > 5% in differential function [6].

Exclusion criteria: secondary giant ureters due to ectopic ureteral opening, neurogenic bladder, posterior urethral valve, or urethral stenosis.

### Surgical approaches

After successful general anesthesia, the patient was in the supine position, with head low and foot high, the bilateral upper limbs posed a "surrender" position, and the bilateral upper limbs slightly opened. All parts of body under pressure were padded with a sponge and fixed with bandages. Disinfecting, draping and urethral catheterization was performed before the following surgery.

#### Single-port-plus-one robot-assisted laparoscopic-modified direct nipple Lich–Gregoir surgery

**Fig. 1:**
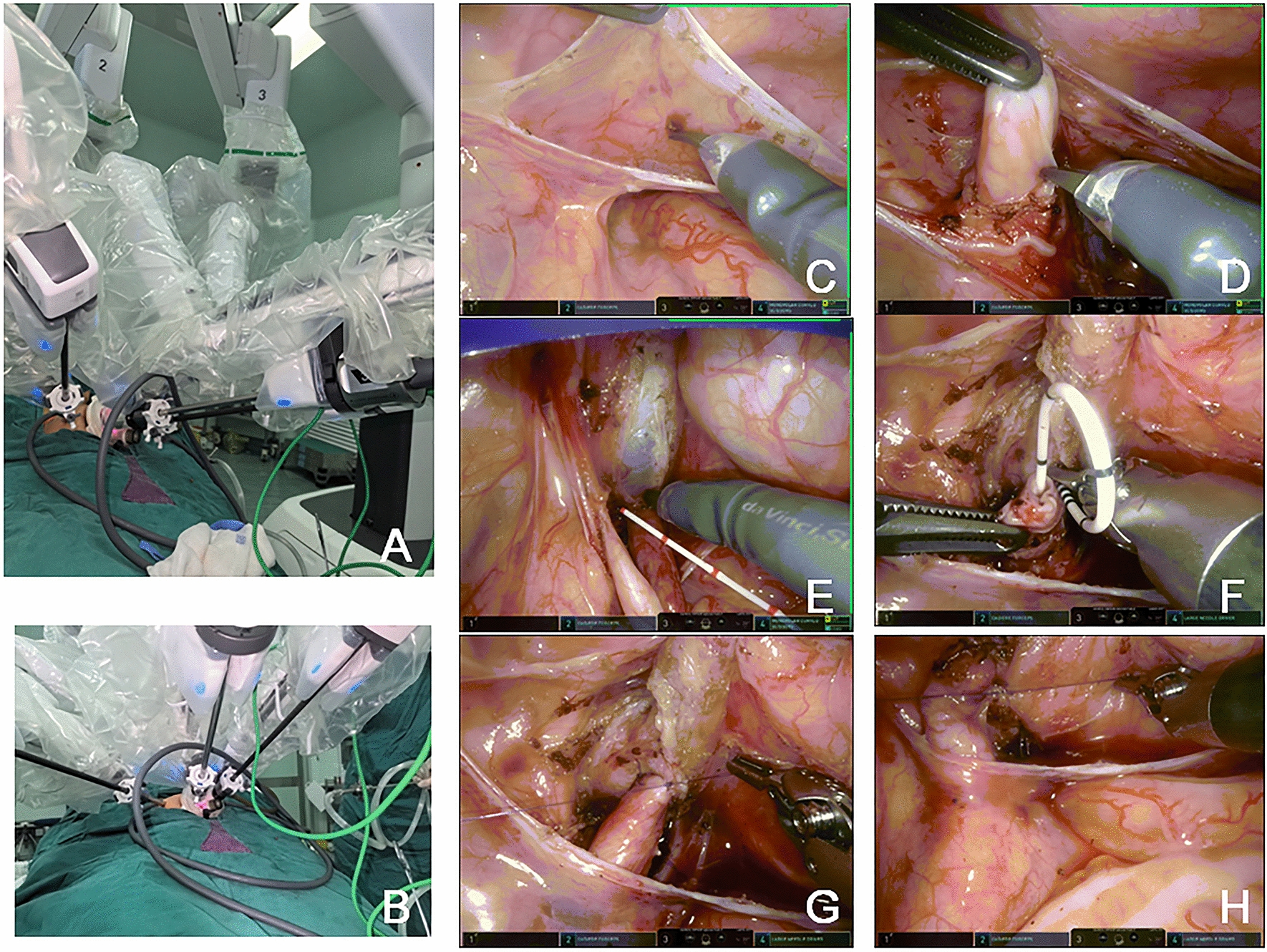
**A** Making a 2.5–3 cm curved incision along the edge of the umbilical to place a quadruple-channel puncture device. The other 8 mm incision was made 6 cm away from 3D camera port III on the left-side of abdomen for inserting robotic operating port II. **B** Then docking with the patient cart in the single-port-plus-one state. **C**, **D** we established a "peritoneal window", in where the vas or uterine artery directly identify from ureter. **E** The direction of detrusor tunnel was designed obliquely. The bladder detrusor was cut and disassembled until bulging of bladder mucosa, the length of cutting according to 5:1 ratio of length to width of the normal ureter. **F**, **G** The overdilation ureter was cut and shaped, put a double J tube into the ureter. The 1.5 cm end of the ureter was placed into the bladder. Sutured the seromuscular layer of ureter and the bladder mucosa at 3,6,9,12 o 'clock position with 6–0 PDS. Finally, 4 stitcheswas done in each quadrants to complete the anastomosis. **H** Apply "down-top" way to complete intermittent incision of cutteddetrusor suture with 4–0 Vicryl

Although the da Vinci system Xi had four robotic arms, we only used three and introduced a quadruple-channel puncture device to improve cosmetic appearance. Making a 2.5–3 cm curved incision along the edge of the umbilical to place a quadruple-channel puncture device, whose four channels would be used to put an 8 mm camera port, an 8 mm right hand operating port and be used as assistant channels. The camera port and right hand  operating port should be placed along the opposite side of single port base to enlanger the distance. Artificial pneumoperitoneum was established with pressure of 10 mmHg  and flow of 4 L/min. The other 8 mm incision was made 6 cm away from umbilicus on the left-side of abdomen for inserting an 8 mm left hand operating port, which will be more flexible for a greater range of motion of surgeon’s left hand. Then docking with the patient cart in the single-port-plus-one state in Fig. 1A, B.

we adopted a new way of creating "peritoneal window". We used a transverse incision to open the peritoneal layer along the surface of posterior bladder wall, dissected the peritoneal layer from posterior bladder wall on both sides, and then used no damage vessel clip to fix the peritoneal layer with the surrounding tissue. In these ways, we established a "peritoneal window", in where the vas or uterine artery directly identify from ureter in Fig. 1C, D.

The direction of detrusor tunnel was designed obliquely and local electrocoagulation marker was generated. Traction sutures were inserted through the abdominal wall above middle of pubic symphysis and sutured bladder muscle layer at the affected ureter side, and then be pulled out from the inserted position. In this way, posterior bladder wall was hanged obliquely. It was convenient for cutting and dissecting detrusor tunnel. Sile saline was poured into to full filling the bladder. The bladder detrusor was cut and disassembled until bulging of bladder mucosa. Stricture segment of ureter was resected, overdilation ureter was cut and shaped if needed, and put a double J tube into the ureter. The ratio of tunnel length to ureteral width was 5:1. The oblique detrusor tunnel was more in line with the physiological direction of the ureter in Fig. 1E. After that, we placed the end of the ureter directly into the bladder. Then we sutured the seromuscular layer of ureter at 1.5 cm away from the end with the bladder mucosa to complete the direct nipple reimplantation in Fig. 1F and G.

Apply "down-top" way to complete intermittent incision of cutted detrusor suture with 4–0 Vicryl. Finally closed the peritoneal layer of the posterior bladder wall and sutured wound. in Fig. 1 H.

#### Traditional laparoscopic Cohen surgery

The pneumovesical approach for Cohen’s cross-trigonal UR was performed as described in the literatures [7–9].

**Fig. 2:**
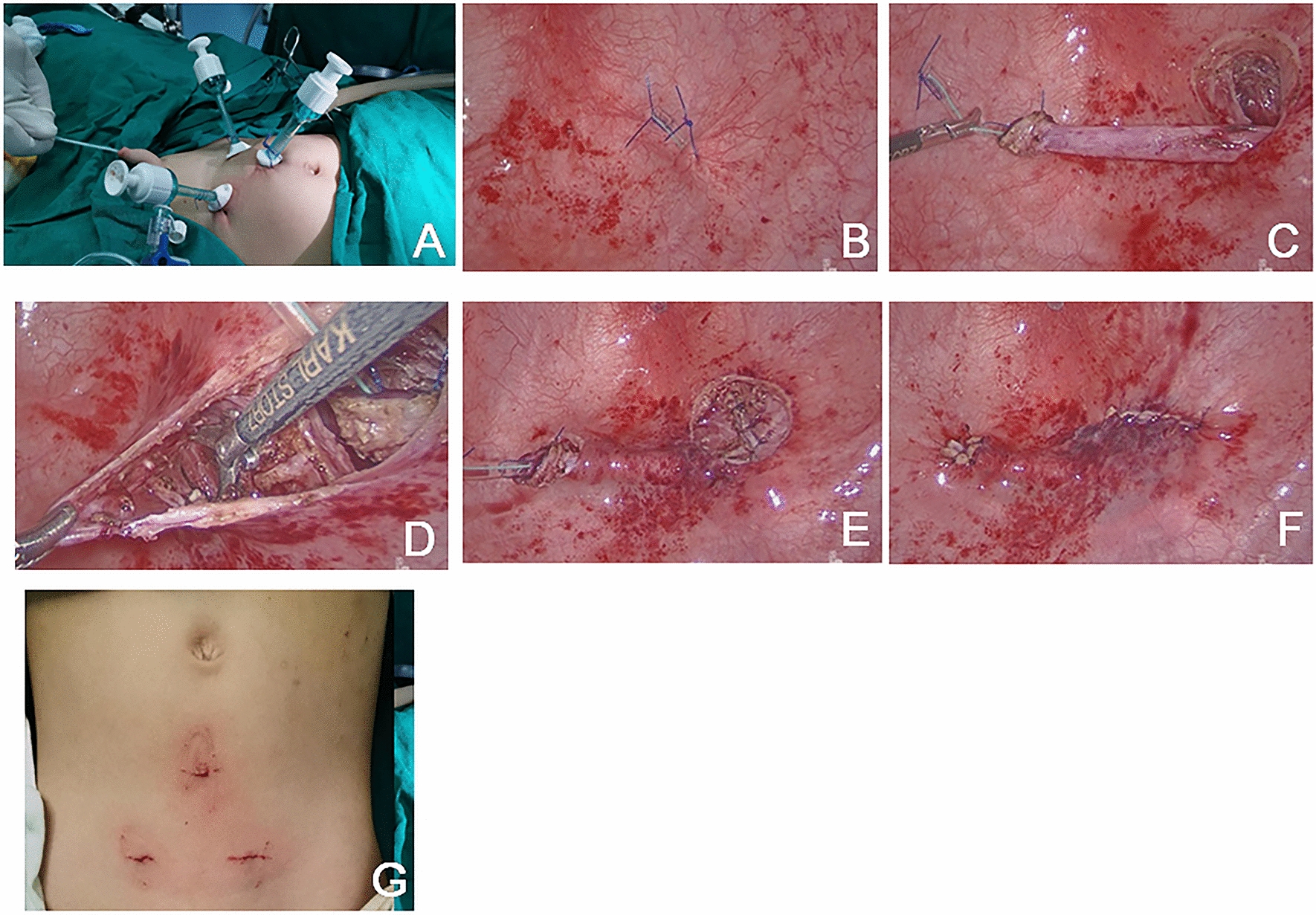
**A** The positions of three trocars in traditional laparoscopic Cohen surgery. **B**, **C** The ureter was mobilized to 2.5–3 cm with circumferential cutting of the mucosal layer surrounding the opening with an electrode hook. **D** Cross-trigonal tunneling is started from the affected side across the bladder trigone to the opposite side, with careful mucosal separation. **E**, **F** The mobilized ureter is then tunneled into the submucosal plane and out to the new orifice. Then finished the anastomosis. **G** The wound of laparoscopic Cohen surgery

A 6F or 8F urethral catheter was inserted into the bladder and filled with normal saline until the bladder was palpably distended. The midline was identified for suprapubic positioning of 3 ports between the umbilicus and top of the pubic bone with the bladder dome situated at the umbilical level along the midline. Then, at 3 cm below the umbilical level, two stitches of 2–0 absorbable sutures were used for percutaneous fixation of the bladder to the anterior abdominal wall. A 5-mm incision was made in the abdomen between the two sutured sites. Then, a 3-mm step port for the camera was inserted. The second and third working ports were inserted 3 cm lateral to the median port on both sides in a similar manner. After the trocars were inserted, the saline was drained, and the bladder was insufflated with CO2 gas at a pressure of 8-12 mmHg and flow rate of 3–4 l/min in Fig. 2A.

Under cystoscopic vision, a 3F ureteral stent was introduced into the urethral opening, inserted into the affected ureter and fixed at the end of the distal ureter with 5–0 absorbable sutures. This facilitates visualization of the extra vesicalureteral pathway. Then, the ureter was mobilized to2.5–3 cm with circumferential cutting of the mucosal layer surrounding the opening with an electrode hook. To minimize injury to the muscularis and surrounding tissues, electrocautery should be localized with the peri-ureteral adventitia. Ureteral tailoring can now be performed as if required in Fig. 2B, C. Cross-trigonal tunneling is started from the affected side across the bladder trigone to the opposite side with careful mucosal separation. At the original ureteral orifice, the defect in the detrusor muscle was closed with interrupted sutures. The remaining mucosal defects were closed with absorbable sutures, and the tube was removed. The ureter was fixed in position with interrupted 5–0 sutures. Anastomosis was performed between the end of the crossed ureter and the bladder mucosa at the neohiatus with 5–0 interrupted sutures dissection in the submucosal plane in Fig. 2D. The new orifice is opened at a site slightly superior to the unaffected ureteral orifice on the other side. The mobilized ureter is then tunneled into the submucosal plane and out to the new orifice, similar to the steps in open surgery in Fig. 2E, F.

### Follow-up

Two months after surgery, patients returned to hospital for cystoscopy to observe the morphology of ureteral orifice, and to remove the double J tube. At 3th and 6th month after the operation, patients took urinary system ultrasound in the outpatient service to identify early complications. One year after the operation, patients returned to the hospital again to evaluate the recovery according to the parameters of MRU, urinary system ultrasound, diuretic renography, DMSA scanning and VCUG examination. At 1_st _year after surgery, every patient underwent cystoscopy to observe the ureteral orifice and 3F catheter was passed through the ureteral orifice to confirm no kicking in ureter.

### Statistical analysis

Continuous variables were compared using Student’s t test, and categorical variables were compared using the chi-square test. Statistical analysis was performed with SPSS Statistics version 20.0 (IBM, Armonk, NY, USA).

## Results

The intro-and postoperative parameters of two group were statistically different. The total operation time of group L is slightly longer than that of group C(Table 2, Group L vs. Group C = 123.58 ± 10.85 min vs. 105.33 ± 8.16 min, *P* < 0.001), but intraperitoneal operation time of two groups were comparable (Table 2, Group L vs. Group C = 101.42 ± 0.85 min vs. 95.58 ± 8.20 min, *P* > 0.05).The blood loss in group L is less than group C (Table 2, Group L vs Group C = 2.42 ± 0.67 ml vs. 4.16 ± 0.58 ml, *P* < 0.001).The ame outcomes were founded in gross haematuria (Table 2, Group L vs. Group C = 16.08 ± 1.44 h vs. 27.33 ± 1.87 h, *P* < 0.001), indwelling catheterization time(Table 2, Group L vs Group C = 2.25 ± 0.45 days vs. 2.75 ± 0.45 days,*** P*** < 0.05), hospitalization (Table 2, Group L vs. Group C = 3.83 ± 0.39 days vs. 4.42 ± 0.51 days, *P* < 0.05). There were no differences in the average follow-up time between two groups (Table 2, *P* > 0.05).

**Table 2:** Operative parameters

Variables^a^	Group L	Group C	*t*	*P*
N (number of patients)	12	12		
Operation time (minutes)				
Total	123.58 ± 10.85	105.33 ± 8.16	4.657	< 0.001
Intraperitoneal	101.42 ± 0.85	95.58 ± 8.20	1.486	0.151
Blood loss (mL)	2.42 ± 0.67	4.16 ± 0.58	− 0.6863	< 0.001
Gross haematuria (hours)	16.08 ± 1.44	27.33 ± 1.87	− 16.471	< 0.001
Indwelling catheterization time(days)	2.25 ± 0.45	2.75 ± 0.45	− 2.708	0.013
Hospitalization (days)	3.83 ± 0.39	4.42 ± 0.51	− 3.130	0.005
Follow-up (months)	14.16 ± 1.75	14.33 ± 1.92	− 0.222	0.862

^a^Values are presented as the mean ± SD unless otherwise stated

The obstruction resolution rate was 100% in both groups. No other postoperative complications were observed except urinary tract infections. Only 2 cases in group C and 1case in group L had urinary tract infections (UTIs) within 2 months postoperatively, which were conservatively treated, and no recurrences were observed after the double J tube was removed.

Pre-operatively, the ureteral diameter (Table 3, Group Lvs. Group C = 18.83 ± 3.21 mm vs. 18.33 ± 3.39 mm, *P* > 0.05). APRPD (Table 3, Group L vs. Group C = 34.92 ± 4.25 mm vs. 34.92 ± 4.87 mm, *P* > 0.05), DRF (Table 3, Group L vs. Group C = 33.75 ± 2.77% vs. 33.92 ± 2.74%, *P* > 0.05) were not significantly different between Group L and Group C. At the 1-year post-operation, there were no distinctively difference founded in the ureteral diameter (Table 3, Group L vs. Group C = 6.83 ± 1.27 mm vs 6.83 ± 1.11 mm, *P* > 0.05). APRPD (Table 3, Group L vs. Group C = 10.08 ± 1.88 mm vs.9.50 ± 2.97 mm, *P* > 0.05), DRF(Table 3, Group L vs. Group C = 37.50 ± 1.31% vs.37.08 ± 1.62%, *P* > 0.05) between Group L and Group C. The delta (difference) between the pre-operative and post- operative values for the ureteral diameter, APRPD, DRF showed no difference in two groups (Table 3, *P* > 0.05).

**Table 3:** Comparison of the preoperative parameters and one-year postoperative parameters in two groups

Variables^a^	Group L	Group C	*t*	*P*
*N* (number of patients)	12	12		
Ureteral diameter (mm),mean ± SD				
Preoperative	18.83 ± 3.21	18.33 ± 3.39	0.371	0.715
First year postoperative	6.83 ± 1.27	6.83 ± 1.11	0.001	1.0
Detal(pre-post,mm), mean ± SD	12.00 ± 2.52	11.5 ± 2.64	0.474	0.640
APRPD (mm), mean ± SD				
Preoperative	34.92 ± 4.25	34.92 ± 4.87	0.001	1.0
First year postoperative	10.08 ± 1.88	9.50 ± 2.97	0.575	0.571
Detal(pre-post,mm), mean ± SD	24.83 ± 3.18	25.41 ± 3.47	− 0.429	0.672
DRF %, mean ± SD				
Preoperative	33.75 ± 2.77	33.92 ± 2.74	− 0.148	0.884
First year postoperative	37.50 ± 1.31	37.08 ± 1.62	− 0.692	0.496
Detal(pre-post,mm), mean ± SD	3.75 ± 1.71	3.16 ± 1.58	0.866	0.396
Obstruction resolution rate	100%	100%		
New VUR rate	0%	0%		

SD, standard deviation; APRPD, anterior-posteior renal pelvic diameter, VUR, vesicoureteral reflux; DRF, defferential renal function

^a^Values are presented as the mean ± SD unless otherwise stated

**Fig. 3:**
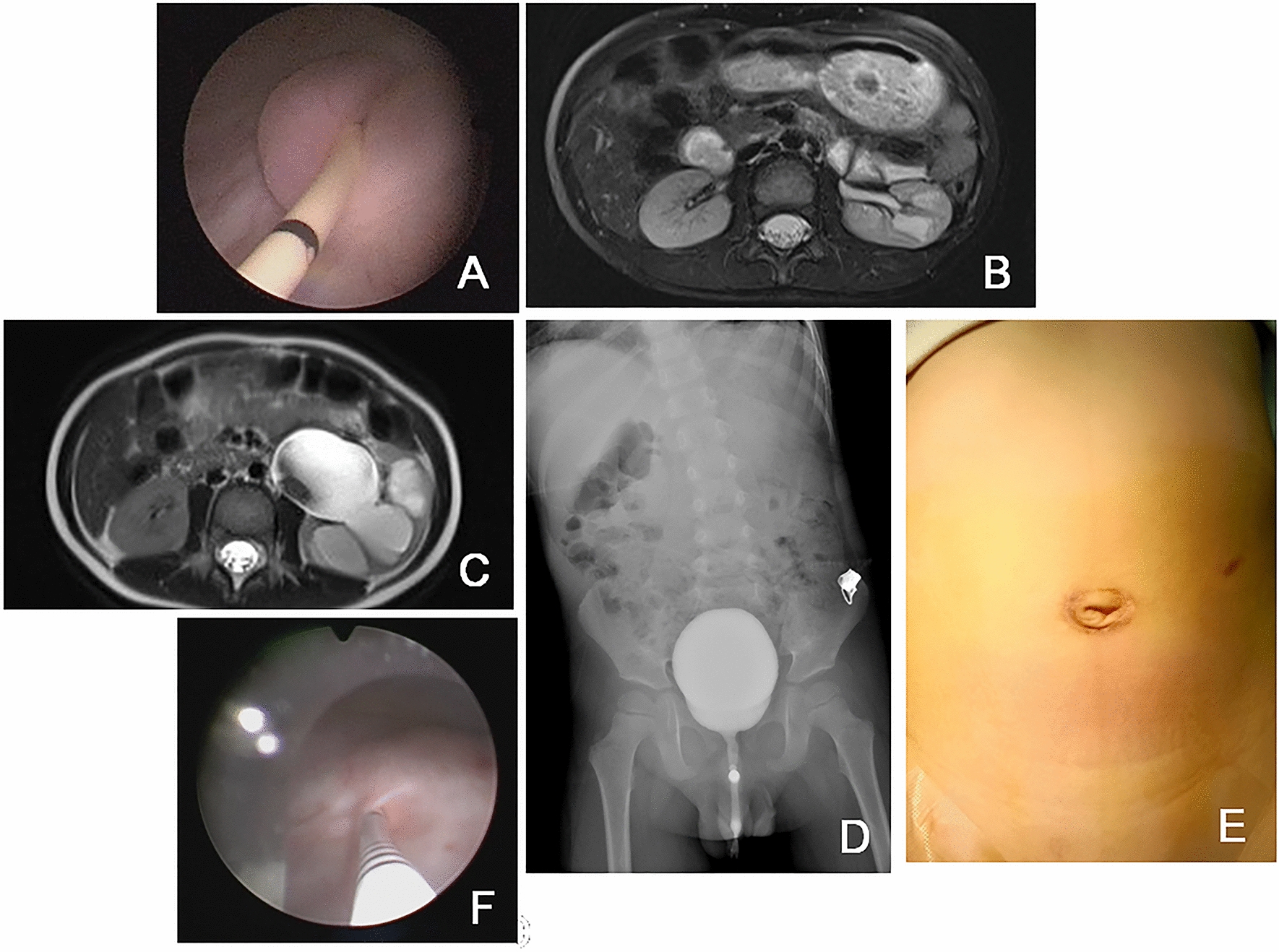
**A** The end of ureter placed into bladder turned into nipple automatically in cystoscopy at 2th month postoperative; **B**, **C** At 1th year postoperative, APRPD and ureteral diameter were significantly reduced compared with those before operation; **D** At 1th year postoperative, there was no newly reflux in VCUG. **E** Cosmetic appearance of wound at 2th month postoperative. **F** 3F catheter was passed through the ureteral orifice smoothly into the ureter which confirming no kicking in ureter in cystoscopy at 1st year post operation

In group L, the end of ureter placed into bladder turned into nipple automatically in cystoscopy at 2th month postoperative Fig. 3A. At the 1-year follow-up, the decreasing of the ureteral diameter and APRPD Fig. 3B, C. At 1th year postoperative, there was no new reflux in VCUG in Fig. 3D. The appearance of wound was cosmetic at 2th month postoperative in Fig. 3E. And 1st year after surgery, 3F catheter was passed through the ureteral orifice smoothly into the ureter which confirming no kicking in ureter in Fig. 3F.

## Discussion

Child is not a miniature of the adult, all trauma in body will be magnified during the growth. Minimally invasive surgery is the main stream of pediatric surgery in the recent years. The Da Vinci robot has features of high-definition, 3D imaging, flexible wrist with 7 degrees of freedom, which are able to overcome the technical bottleneck of laparoscopic surgery and suitable for more complex reconstructive surgery. These advantages have expanded its application in the field of pediatric surgery, especially in pediatric urinary tract reconstruction [10].

With the advance of laparoscopic technology at the end of the twentieth century, surgeons strived to achieve ERAS and make the wound more aesthetic at the same time. In the mid-twenty-first century, trans-umbilical single-site laparoscopic surgery was generated, which concentrates 3–4 laparoscopic wounds around the umbilicus to hide wounds and make the appearance more cosmetic. However, the linear laparoscopic instruments placed in a single hole had a significant chopstick effect during the operation, which made the traction and suture more difficult. The flexible wrist of the da Vinci robot, which can rotate angle more than 540 degrees, is able to overcome the deficiency of laparoscopic instruments in trans-umbilical single-site laparoscopic surgery. Since reported in 2019, trans-umbilical single-port robot-assisted surgery had become a new trend in the development of minimally invasive surgical techniques.

However, the range of the single-port robot-assisted surgery was limited due to the difficulty of the technique. The motion range of robot arm was limited by single-port, which could only allow the advance and retreat. The surgeon could not reach multiple quadrants of the vision and should had sophisticated single-port laparoscopic surgery experience in the previous work. The defects restricted the usage of the technique, which was mainly used in relatively simple surgery such as pyeloplasty and nephrectomy in pediatric. The Journal of Robotic Surgery was not recommended the SP robot to be performed in the infants especially in low weight infants, due to arm collision and the inability to advance and see the four quadrants [11]. To solve the problem we apply the single-port-plus-one robot technique, which could maintain the advantages of robot surgery in ERAS and appearance,largely decrease the difficulty and broadened the scope of applications, particularly in urinary tract and bile duct reconstructive surgery [12, 13]. In this study, we found the total operation time in group L is slightly longer than that in group C, which may due to the docking and installing of the robot arms, but intraperitoneal operation time of two groups was comparable. The postoperative parameters included blood loss, gross haematuria time, indwelling catheterization time and hospitalization time in group L is shorter than group C.

Cohen procedure has been comfired effective in ureteral reimplantation for decades. It can be implemented in open or vesicoscopic approach.Vesicoscopic Cohen closely replicated the open procedure and was reported to have equally good results. Nowdays, robot-assisted Cohen vesicoscopic procedure has been reported in literature,which fell in conclusion that it was feasible and also could replicate vesicoscopic procedure by experienced robotic surgeon [14]. There were many opinions in comparison of the Lich–Gregoir and the Cohen procedure in literatures [15, 16]. Our department did not implement the robotic assisted Cohen vesicoscopic procedure, but we modified the robotic assisted laparoscopic Lich–Gregoir procedure, so we compared it with vesicoscopic Cohen procedure in this acticle.

In Cohen operation, neohiatus of ureter was in the original site and new ureteral orifice was opened above the opposite one through the submucous tunnel. The length of submucosal tunnel length is limited by the width of the bladder trigone, and the common ureteral tailoring manner is technically demanding with the intravesical approach. The Cohen technique was reported to be effective in anti-refluxing, but it seemed to change the normal physiological structure of the bladder and ureter. The submucous tunnel was transverse which was impossible to the treatment of ureteral disease through ureteroscopy when the patients grow up. In 2022, we developed trans-vesicoscopic Politano-Leadbetter technique in ureteral reimplantation and compared it with Cohen technique [17]. We found the importance of preserving the natural direction of the ureters in this new technique, which is more conducive to urine excretion, recovery of renal function. And at 1th year post operation, smoothly retrograded placement of 3F ureteral stent under ureteroscopy also confirm the physiological anatomical structure of ureter. In 2011, we developed direct nipple implantation technique. Later in follow up, the end of ureter placed in bladder turned outward into nipple automatically, which strengthened the anti-reflux effect and prevented the occurrence of postoperative VUR.

Lich–Gregoir technique was primitively used in the treatment of VUR. In 2006, Ansari et al. reported the first 3 cases of laparoscopic ureteral extravesical reimplantation by the Lich–Gregoir technique in the treatment of POM [18]. From then on, similar cases had been reported [19–21]. In all cases, there were a decrease in ureteral and upper tract dilatation, as well as improved drainage [22, 23]. Inspired by the previous research, we modified the Lich–Gregoir technique by obliquely produced detrusor tunnel to ensure the physical direction of ureter and combined it with direct nipple reimplantation in the treatment of POM. Main concern in the Lich–Gregoir technique is the risk of voiding dysfunction. The main portion of the pelvic plexus are located about 1.5 cm dorsal and medial to the ureterovesical junction and they travele just outside Waldeyer's sheath, leaving a safe area for surgical dissection under the sheath [24, 25].In our modified technique, detrusor tunnel was designed obliquely outside and the ureter was dissected carefully within Waldeyer's sheath. And voiding dysfunction was rare certainly in unilateral cases. We didn’t encountered the problem in the group L. The results come out to comparable with the tradition Cohen technique.

There are many limitations in this study.Such as the quantity is limited, the follow-up time is insufficient, the cases are all unilateral. As this article is the first report about robotic assisted laparoscopic Lich–Gregoir reimplantation in children, we focus on introduced the surgrical technique and experience. Further improvement will be made in future studies.

## Conclusion

Single-port-plus-one robot-assisted laparoscopic-modified Lich–Gregoir direct nipple approach and traditional laparoscopic Cohen are both dependable techniques for ureteral reimplantation in the treatment of pediatric primary obstructive megaureter. With higher accuracy, single-port-plus-one robot-assisted laparoscopic technique has advantages in achieving enhanced recovery and concealing incision. Since Lich–Gregoirc preserves the physiological direction of the ureter and direct nipple reimplantation enhances the effect of anti-refluxing, this technique is favorable for being promoted and applied in robot surgery.

## Data Availability

The data that support the findings of this study are available on request from the corresponding author.

## Abbreviations

APRPD: Anterior–posterior renal pelvic diameter
DRF: Differential renal function
DMSA: Dimercaptosuccinic acid
MIS: Minimally invasive surgical
MRU: Magnetic resonance urography
POM: Primary obstructive megaureter
UR: Ureteral reimplantation
UTIs: Urinary tract infections
UTDS: Urinary tract dilation risk stratification
VCUG: Voiding cystourethrogram
VUR: Vesicoureteral reflux
ERAS: Enhanced recovery after surgery
